# A Meta-Analysis of Probiotic Efficacy for Gastrointestinal Diseases

**DOI:** 10.1371/journal.pone.0034938

**Published:** 2012-04-18

**Authors:** Marina L. Ritchie, Tamara N. Romanuk

**Affiliations:** Department of Biology, Dalhousie University, Halifax, Nova Scotia, Canada; Charité, Campus Benjamin Franklin, Germany

## Abstract

**Background:**

Meta-analyses on the effects of probiotics on specific gastrointestinal diseases have generally shown positive effects on disease prevention and treatment; however, the relative efficacy of probiotic use for treatment and prevention across different gastrointestinal diseases, with differing etiology and mechanisms of action, has not been addressed.

**Methods/Principal Findings:**

We included randomized controlled trials in humans that used a specified probiotic in the treatment or prevention of Pouchitis, Infectious diarrhea, Irritable Bowel Syndrome, *Helicobacter pylori*, *Clostridium difficile* Disease, Antibiotic Associated Diarrhea, Traveler's Diarrhea, or Necrotizing Enterocolitis. Random effects models were used to evaluate efficacy as pooled relative risks across the eight diseases as well as across probiotic species, single vs. multiple species, patient ages, dosages, and length of treatment. Probiotics had a positive significant effect across all eight gastrointestinal diseases with a relative risk of 0.58 (95% (CI) 0.51–0.65). Six of the eight diseases: Pouchitis, Infectious diarrhea, Irritable Bowel Syndrome, *Helicobacter pylori*, *Clostridium difficile* Disease, and Antibiotic Associated Diarrhea, showed positive significant effects. Traveler's Diarrhea and Necrotizing Enterocolitis did not show significant effects of probiotcs. Of the 11 species and species mixtures, all showed positive significant effects except for *Lactobacillus acidophilus*, *Lactobacillus plantarum,* and *Bifidobacterium infantis*. Across all diseases and probiotic species, positive significant effects of probiotics were observed for all age groups, single vs. multiple species, and treatment lengths.

**Conclusions/Significance:**

Probiotics are generally beneficial in treatment and prevention of gastrointestinal diseases. Efficacy was not observed for Traveler's Diarrhea or Necrotizing Enterocolitis or for the probiotic species *L. acidophilus*, *L. plantarum,* and *B. infantis*. When choosing to use probiotics in the treatment or prevention of gastrointestinal disease, the type of disease and probiotic species (strain) are the most important factors to take into consideration.

## Introduction

The efficacy of using probiotics in the prevention and treatment of gastrointestinal diseases has received considerable attention in recent years [1]–[5]. In western civilization, there has been an increase in gut-related health problems, such as autoimmune and inflammatory diseases [6]. Changes in the gut flora have emerged as a leading mechanism for the increased prevalence of certain gastrointestinal diseases [6]–[8]. Due to improved hygiene and nutrition, the western human diet contains several thousand times less bacteria than pre-industrialized diets [6], [9]. This is partially due to the use of processed and sterile foods which contain artificial sweeteners and preservatives, rather than fresh fruits and vegetables [10], or foods containing important microbes for anti-inflammatory processes [11], [12].

Probiotics, products or preparations containing sufficient amounts of viable microorganisms to alter a host's microflora communities [13], are thought to exert beneficial effects by providing protective barriers, enhancing immune responses, and clearing pathogens in the gastrointestinal tract [14]–[16]. Meta-analyses or clinical trials on the efficacy of probiotics have been conducted for a number of common gastrointestinal diseases including Irritable Bowel Syndrome (IBS) [5], *Helicobacter pylori* infection (HPP) [3], Necrotizing Enterocolitis (NEC) [17], Pouchitis (Pouch) [18], Antibiotic Associated diarrhea (AAD) [19], *Clostridium difficile* Disease (CDD) [20], Infectious diarrhea (ID) [2], and Travellers diarrhea (TD) [2]. These studies have shown that probiotics have significant effects on the prevention (e.g. [2]) and treatment (e.g. [20]) of gastrointestinal disease. While numerous meta-analyses have been performed on the use of probiotics in the prevention and treatment of specific diseases (e.g. [3], [5], [17]), to our knowledge, a meta-analysis comparing the efficacy of probiotics across various diseases has not been conducted. Probiotics have been used to prevent and treat a wide range of GIT diseases. The GIT diseases considered here can be grouped into three groups based on symptomology: 1) production of diarrhea: AAD, CDD, ID, TD, 2) the destruction or inflammation of tissues in the stomach, large intestine, ileal reservoir, or bowel: NEC, Pouch, and HPP, 3) abdominal pain, flatulence, and irregular bowel movements: IBS. The etiology of re-occurring and chronic inflammation in the gastrointestinal tract is not definitive [21]. Nevertheless, evidence suggests that an imbalance of intestinal bacteria may commence and perpetuate the inflammation that characterizes the gastrointestinal diseases related to chronic and re-occurring inflammation [22]–[24]. Furthermore, pathogenic bacteria can invade tight junctions between epithelial cells and disturb the barrier function of the gut, resulting in translocation of pathogenic bacteria that leads to an inflammatory immune response [25].

Previous studies have shown probiotic efficacy in treating inflammation-related, diarrhea-related, and IBS symptoms [26], [27], [28]. The primary mechanisms of action of probiotics are modification of the gut microflora [6], stabilization of the indigenous microflora [29], reductions in the duration of retrovirus shedding [30], and a reduction in increased gut permeability which is caused by retrovirus infection [31]. In diarrhea-related diseases, probiotics may induce a general immune response, in addition to increasing IgA antibodies against rotavirus [32], [33]. In inflammatory-related disease, probiotics are thought to decrease disease activity and promote remission [34]. Reductions in inflammation are thought to occur by decreasing pathogenic bacterial growth through the enhancement of barrier functions which prevents the invasion of tight junctions, by lowering gut pH, and by stimulating non-specific and specific immune responses [34]. IBS has been correlated with a lower amount of *Lactobacilli* and *Bifidobacterium* colonies and an increase in anaerobic *Clostridium spp*. which has taken place of anaerobic *Bifidobacterium* spp. and *Bacteriodes spp.* [35], [36]. Therefore, there are links between humans consuming lactose and sucrose with an onset of IBS [35], which is thought to be caused by providing the pathogenic microbial population with a nutritional source [35]. As a result, probiotics such as *L. plantarum* [37], and *Enterococcus faecum* [38] have been used to treat IBS because they compete for the same food source. Not all these mechanisms of action will apply to all the GIT diseases considered here, thus by comparing probiotic efficacy across diseases it may be possible to assess the specific functional responses by which probiotics are operating.

Here we report on a meta-analysis designed to determine whether probiotics are more or less effective in the prevention and treatment of eight different gastrointestinal diseases across 11 species or species mixtures of probiotics. We further assessed whether patient age, dose, length of treatment, and single vs. multiple probiotic species affect efficacy as previous studies have shown differences in probiotic efficacy based on these factors.

## Methods

### Objectives

The objectives of this meta-analysis were to: (i) determine the overall effect of probiotics on diseases of the gastrointestinal tract that have previously been shown to be affected by probiotics, (ii) determine whether certain diseases respond to probiotics more than others (iii) determine whether different species and species combinations differed in their overall effect size, and to (iv) determine whether efficacy differs based on dosage, length of treatment, and age group.

### Search Strategy and Study Selection

The supporting PRISMA checklist is available as supporting information; see Checklist S1. We conducted a literature search for randomized controlled efficacy trials in humans for probiotics used in the prevention and treatment of gastrointestinal disease. We searched Pubmed (January 1970 to January 2011), Medline (January 1970 to January 2011), Google Scholar (January 1970 to January 2011), Embase (January 1970 to January 2011), Biological Abstracts (January 1970 to January 2011), and Science Direct (1970 to January 2011). Search terms included: probiotics, probiotic meta-analysis, Gastrointestinal disease, Diarrhea, *Helicobacter pylori,* Pouchitis, Antibiotic Associated Diarrhea, Irritable Bowel Syndrome, Travellers Diarrhea, *Clostridium difficle* Disease, Necrotising Enterocolitis, Infectious Diarrhea, yogurt, *Lactobacillus*, *Bifidobacterium*, *Saccharomyces*, *Streptococcus*, *Enterococcus,* randomized control trials, controlled trials, placebo, and control. Searches were not restricted by language and secondary searches were done by reference lists, authors and reviews (e.g. Appendix S1). Excluded trials included case reports or case series, trials of unspecified probiotics, trials on prebiotics, trials with inconsistent outcome measures, trials with no specific disease being studied, and trials on animals other than humans. Eligibility criteria included: randomized controlled trials published in peer-reviewed journals, humans with gastrointestinal disease (AAD, CDD, HPP, IBS, ID, NE, Pouch, TD), and studies that compared probiotic therapy with placebo or no therapy. After excluding trials that did not fit the criteria, a total of 84 suitable trials were identified for analysis spanning 10,351 patients, 11 probiotic species or mixtures, and eight diseases. Of the 84 suitable trials that are analyzed in this meta-analysis, 79 have been cited in meta-analyses on their specific disease [1]–[5], [13], [17], [18], [ and 20].

### Outcome Assessment

The primary outcome assessed was prevention in overall symptoms or treatment of the gastrointestinal diseases. Here we use prevention and treatment interchangeably when discussing the effects of probiotics across all diseases as for some diseases (i.e. CDD; [20]) probiotics are effective in both prevention and treatment. For other diseases, probiotics have only shown to have efficacy in either prevention or treatment. For example, probiotics are used in the prevention of diarrhea [39] and in the treatment of IBS [4]. The outcomes for the efficacy of the eight gastrointestinal diseases are shown in Table 1.

**Table 1:** List of primary outcomes for the eight gastrointestinal diseases analyzed in this meta-analysis.

Disease	Outcome
Antibiotic Associated Diarrhea (AAD), Traveller's Diarrhea (TD), and Infectious Diarrhea (ID)	The primary outcome for AAD, TD, and ID is defined as diarrhea (3 loose stools/day for at least 2 days or 5 loose stools/48 h) within 2 months of antibiotic exposure.
*Clostridium difficile* Disease (CDD)	The primary outcome of CDD is defined as a new episode of diarrhea associated with a positive culture or toxin (A or B) assay within 1 month exposure to antibiotics. The outcome of prevention of CDD is a new episode of *C. difficle* positive diarrhea within 1 month of a previous CDD episode.
Irritable Bowel Syndrome (IBS)	The primary outcome measures was the improvement in overall symptoms as defined by the presence or absence of the following physical symptoms: pain, flatulence, bloating, anxiety, and quality of life or the change in symptom scores from baseline.
*Helicobacter pylori* (HPP)	The primary outcome was the improvement of *H. pylori* eradication rates reducing side effects with probiotics.
Necrotizing Enterocolitis (NEC)	The primary outcome of efficacy of probiotic supplementation in prevention of stage 2 or greater Necrotizing Enterocolitis, and safety in terms of blood culture-positive septis and any other adverse events reported by investigators.
Pouchitis (Pouch)	The primary outcome of efficacy of probiotic supplementation was for the treatment of Pouchitis with no relapse.

### Data extraction and risk of bias

From each paper we extracted information related to disease, probiotic species, the dose amount, treatment length, age group, number of trials, number of patients receiving the probiotic or the control, and the number of patients that improved following probiotic/control. A few studies had multiple probiotic treatments with a common control group and were analyzed separately.

One author (Ritchie) independently reviewed and assessed inclusion criteria and quality of trials. Each included study was assessed using a 5-point Jaded scale [40] based on randomization, concealment of allocation, blinding of investigators, including outcome assessors, and completeness of follow-up. Inconsistencies were resolved by discussion of the authors. Weights for the meta-analysis are based on sample sizes.

### Data synthesis and statistical analysis

A random effects meta-analysis was conducted with inverse variance weighting using the software MIX version 2.0 Pro [41]. For each paper the relative risk ratio (RR), which is the ratio of the probability of the event occurring in the probiotic treatment versus the control group [42], was calculated along with 95% confidence intervals, and summary statistics. Overall RR, heterogeneity (I^2^), z-values, and p-values were computed across all studies and for each comparison. If significant heterogeneity (I^2^) occurred (p<0.05) the studies were analyzed using a random effects model with a pooled relative risk. If the studies were not significant (p>0.05) they were analyzed using a fixed effect model with a pooled relative risk. Effect sizes (RR values) that were <1 favoured the probiotic while effect sizes that were >1 favoured the placebo. If the 95% confidence intervals of effect sizes do not overlap, the RR is considered significantly different. Publication bias was assessed by funnel plot asymmetry [43]. Risk ratios were plotted against the standard error of the risk ratio of each study to identify asymmetry in the distribution of trials. Potential publication bias is suggested when there is a gap in the funnel plot. Begg's regression test was also used to assess potential publication bias [44]. The Fail safe N-Method defined as, “the number of new, unpublished, or un-retrieved non-significant or “null result” studies that would be required to exist to lower the significance of a meta-analysis to some specified level” [45] was also used for bias analysis.

Six different factors were included in the meta-analysis: the disease treated with probiotics (AAD, CDD, IBS, ID, TD, NEC, Pouch, and HPP), the type of probiotic used (VSL#3, LGG, S. *boulardii*, *B. infantis, L. acidophilus*, *L. casei*, *C. butyricum*, *E. faecum*, *L. plantarium*, *B. lactis* and L. *acidophilus* combined with *B. infantis*, the dose of the probiotic (1–9×10^11^, 10^12^ CFU/day; 1–5.5×10^6^, 10^7^, 10^8^ CFU/day; 1–9×10^9^ CFU/day; 1–5×10^10^ CFU/day), the amount of time the probiotic was administered for (9–240 weeks, 5–8 weeks, 3–4 weeks, 1–2 weeks), the age group of the subjects receiving probiotics (infants (0–3 yrs), children (3≤18 yrs), adults(>18 yrs)) and single versus multiple species of probiotics (Materials S1).

## Results

### Overview of included studies

**Figure 1:**
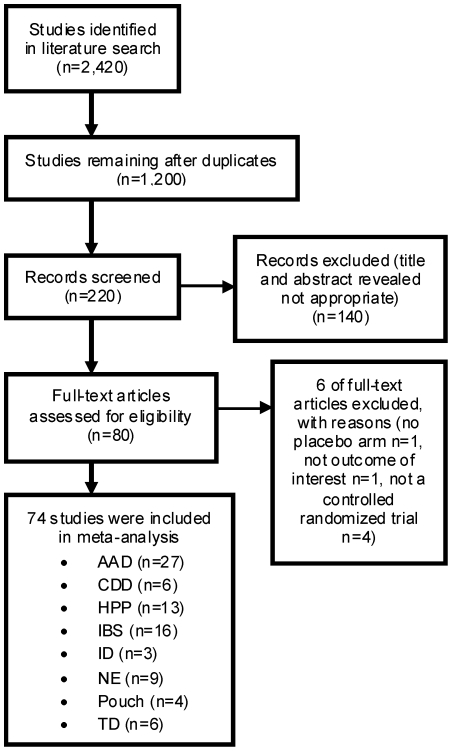
PRISMA (Preferred Reporting Items for Systematic reviews and Meta-Analyses) flow diagram showing an overview of the study selection process.

**Figure 2:**
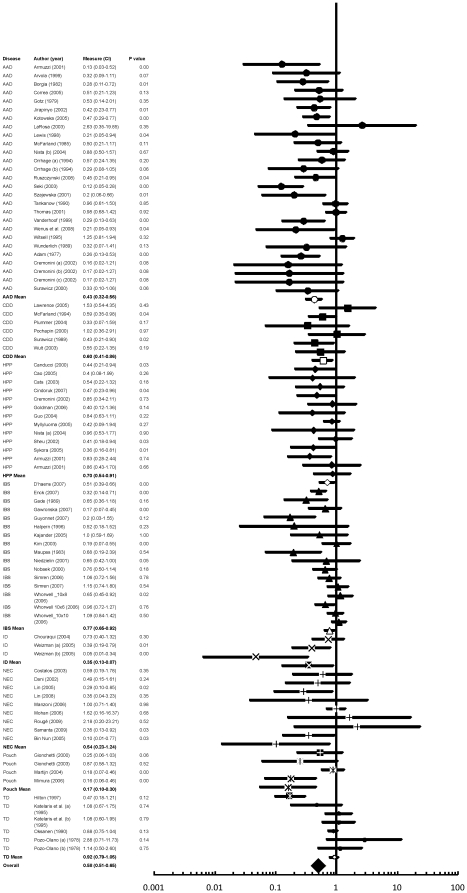
The effect size (risk ratio) for the overall effects of probiotics in the prevention and treatment of gastrointestinal (GI) diseases including the 95% confidence intervals. The diseases: Antibiotic associated diarrhea (AAD), *Clostridium difficile* disease (CDD), *Helicobacter pylori positive* (HPP), Irritable bowel syndrome (IBS), Infectious diarrhea (ID), Necrotizing Enterocolitis (NEC), Traveller's diarrhea (TD), and Pouchitis are labelled as well as the mean effect sizes for each disease. The author, date, measure (risk ratio (95% CI), and p value are shown. Risk ratios below one favor the probiotic while risk ratios above one favor the placebo.

The literature search yielded 2,420 citations, of which 220 were screened and 80 were assessed for eligibility. Of these, 6 were excluded for various reasons (Figure 1), leaving 74 studies that met the inclusion criteria. Therefore, 84 peer-reviewed trials were included in the meta-analysis. All trials included in this meta-analysis had a Jaded quality score of 3 or more, except for 4 of them which had a score of 2 due to unavailable information (Materials S1). The median number of patients per trial was 88.5 ranging from 15–756. In total, 10,351 subjects were included in the studies. Of the 84 trials, 31 (37%) showed a significant reduction of GI diseases in the probiotic treated patients compared with the control patients. 53 trials did not reject the null hypothesis of no difference in the incidence of GI disease for probiotic verses controls. The pooled estimate of efficacy of probiotics in prevention or treatment of disease yielded a relative risk of 0.58 (95% CI 0.51–0.65; p<0.001) and a heterogeneity (I^2^) of 61.24% (95% CI 51–69; X^2^ p<0.001) showing that across all diseases and probiotic species, probiotics were effective in the treatment and prevention of GI diseases (Figure 2).

### Effect by types of disease

**Figure 3:**
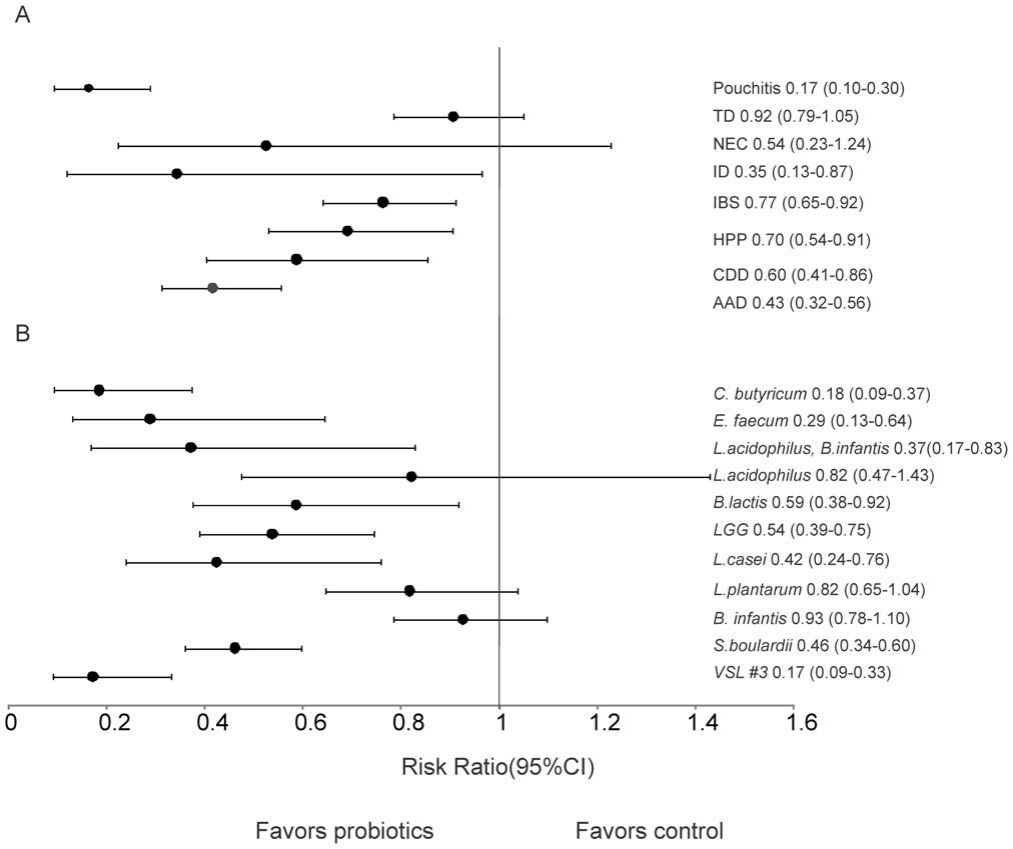
The effect size (risk ratio) for gastrointenstinal diseases and for probiotic species. (A) The effect size including the 95% confidence intervals for the total events of Antibiotic associated diarrhea (AAD), *Clostridium difficile* disease (CDD), *Helicobacter pylori positive* (HPP), Irritable bowel syndrome (IBS), Infectious diarrhea (ID), Necrotizing Enterocolitis (NE), Traveller's diarrhea (TD), and Pouchitis during which probiotics were taken. (B) The effect size including 95% confidence intervals for the type of probiotic species that were used to treat and prevent gastrointestinal disease. The species that were used were *VSL#3*, *Lactobacillus rhamnosus GG* (LGG), *Saccromyces boulardii*, *Bifidobacterium infantis, Lactobacillus acidophilus*, *Lactobacillus casei*, *Clostridium butyricum*, *Enterococcus faecum*, *Lactobacillus plantarium*, *Bifidobacterium lactis* and *Lactobacillus acidophilus* combined with *Bifidobacterium infantis*. Risk ratios below one favor the probiotic while risk ratios above one favor the placebo.

Within the eight diseases considered, Pouchitis (n = 4; RR = 0.17; 95% CI 0.10–0.30), AAD (n = 27; RR = 0.43; 95% CI 0.32–0.56), ID (n = 3; RR = 0.35; 95% CI 0.13–0.97), IBS (n = 16; RR = 0.77; 95% CI 0.65–0.92), HPP (n = 13; RR = 0.70; 95% CI 0.54–0.91), and CDD (n = 6; RR = 0.60; 95% CI 0.41–0.86) yielded significant effect sizes (Figure 3a). Significant effect sizes were not observed for probiotics for the diseases TD (n = 6; RR = 0.92; 95% CI 0.79–1.05) and NEC (n = 9; RR = 0.54; 95% CI 0.23–1.24) (Figure 3a). Efficacy for Pouchitis was significantly greater than for TD, IBS, HPP, CDD, and AAD. When comparing the diseases that cause diarrhea to those that cause tissue damage/inflammation and to IBS, no significant effect was found (Figure 3a).

### Effect by probiotic species

Across all diseases, eight species yielded significant effect sizes including: VSL #3 which contains viable lyophilized bacteria of four species of *Lactobacillus* (*L. casei, L. plantarum, L. acidophilus*, and *L. delbrueckii* subsp. *bulgaricus*), three species of *Bifidobacterium* (*B. longum, B.breve*, and *B. infantis*), and one species of *Streptococcus salivarius* subsp. (n = 3; RR = 0.17; 95% CI 0.09–0.33), *E. faecium* (n = 2; RR = 0.29; 95% CI 0.13–0.64), *C. butyricum* (n = 2; RR = 0.18; 95% CI 0.09–0.37), *L. acidophilus* combined with *B. infantis* (n = 3; RR = 0.37; 95% CI 0.17–0.83), *B. lactis* (n = 3; RR = 0.59; 95% CI 0.38–0.92), *LGG* (n = 14; RR = 0.54; 95% CI 0.39–0.75), *L. casei* (n = 3; RR = 0.42; 95% CI 0.24–0.76) and *S. boulardii* (n = 11; RR = 0.46; 95% CI 0.34–0.60) (Figure 3b). The other three probiotic species (*L. acidophilus, L. plantarum,* and *B. infantis*), did not show significant efficacy (Figure 3b). *S. boulardii* showed significantly higher efficacy than *L. plantarum* and *B. Infantis*. *C. butyricum* had significantly higher efficacy from the species *L. plantarum*, *L. acidophilus*, *LGG*, *L. plantarum* and *B. Infantis.* VSL #3 had significantly higher efficacy than the species *S. boulardii*, *B. infantis*, *L. plantarum*, *LGG*, *B. lactis*, and *L. acidophilus* (Figure 3b). As *L. acidophilus* is one of the most common probiotics we further considered whether differences in efficacy were observed based on particular strains. We found that when analyzed alone, *L. acidophilis* LB did show significant efficacy (RR = 0.40 95% CI 0.20–0.82) and *L. acidophilus* with no strain specified did not have a significant effect (RR = 1.17 95% CI 0.85–1.62).

### Effects of age

**Figure 4:**
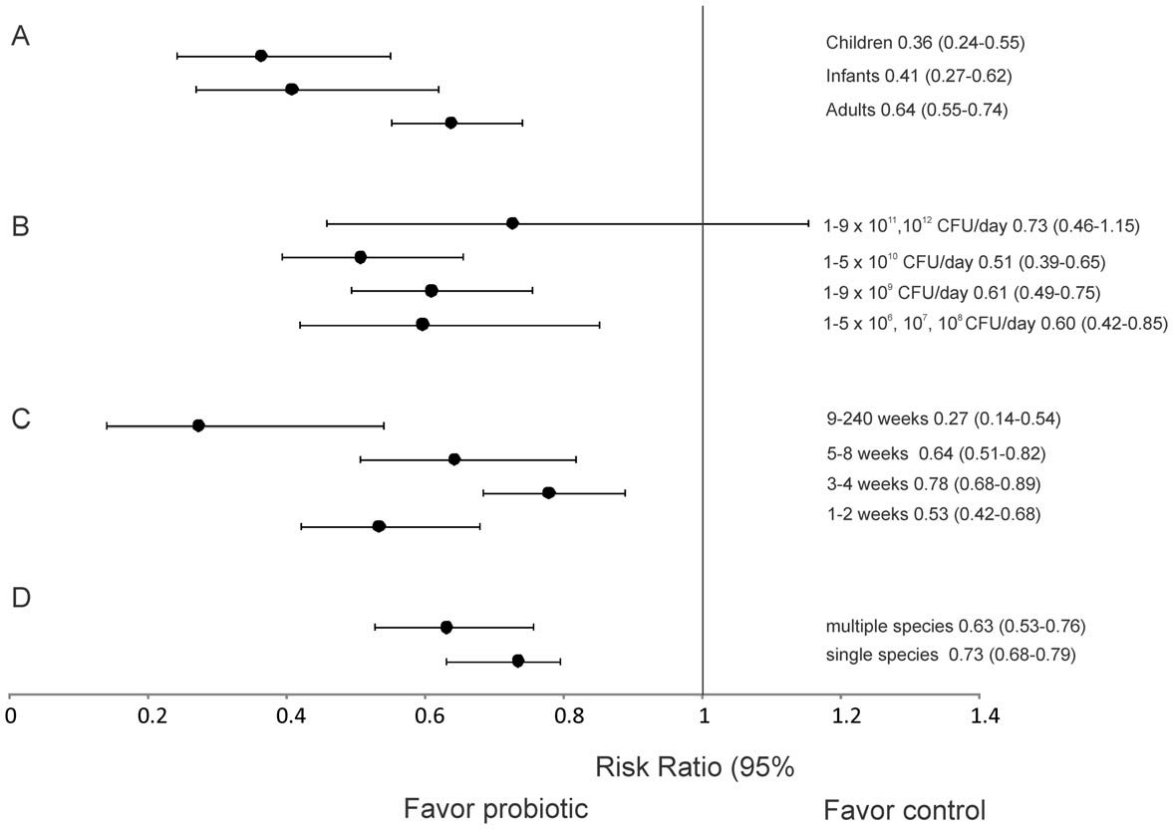
The effect size (risk ratio) for the subgroup analyses on age groups, dose, treatment length and multiple or single probiotic species. (A) The effect size including the 95% confidence intervals for the age groups that had taken the probiotic vs. the controls. Age groups included were: adults (>18 yrs), children (3≤18 yrs) and infants (0–3 yrs). (B) The effect size including the 95% confidence intervals for dose of probiotic. The doses that were included were: 1–9×10^11^, 10^12^ CFU/day; 1–5.5×10^6^, 10^7^, 10^8^ CFU/day; 1–9×10^9^ CFU/day; 1–5×10^10^ CFU/day. (C) The effect size including the 95% confidence intervals for treatment length. Treatment lengths that were included were: 1–2 weeks, 3–4 weeks, 5–8 weeks and 9–240 weeks. (D) The effect size including the 95% confidence intervals for multiple or single species of probiotics. Probiotics that contain more than one species were considered multiple species, while probiotics only administered as one species were considered single species. Risk ratios below one favor the probiotic while risk ratios above one favor the placebo.

Across all diseases and probiotic species, significant efficacy was observed for all of the age groups studied (infants (n = 9; RR = 0.41; 95% CI 0.27–0.62, children (n = 14; RR = 0.36; 95% CI 0.24–0.55), and adults (n = 53; RR = 0.64; 95% CI 0.55–0.74) (Figure 4a). None of the age groups were significantly different from each other (Figure 4a).

### Effects of Dose

Across all diseases and probiotics species, significant efficacy was observed for three doses: 1–5×10^10^ CFU/day (n = 20; RR = 0.51; 95% CI 0.39–0.65), 1–5.5×10^6^, 10^7^, 10^8^ CFU/day (n = 12; RR = 0.60; 95% CI 0.42–0.85), and 1–9×10^9^ CFU/day (n = 25; RR = 0.61; 95% CI 0.49–0.75) (Figure 4b). One dose (1–9×10^11^, 10^12^ CFU/day, n = 7; RR = 0.73; 95% CI 0.46–1.15) did not have significant efficacy (Figure 4b). None of the dose groups were significantly different from each other (Figure 4b)

### Effect of treatment length probiotic was administered

Subgroup analysis for length of treatment showed significant efficacy for all of the four groups; 1–2 weeks (n = 30; RR = 0.53; 95% CI = 0.42–0.68), 3–4 weeks (n = 21; RR = 0.78; 95% CI 0.68–0.89), 5–8 weeks (n = 18; RR = 0.64; 95% CI = 0.51–0.82), and 9–240 weeks (n = 7; RR = 0.27; 95% CI 0.14–0.54). The longest treatment period (9–240 weeks) had significantly higher efficacy than the 3–4 week treatment length group (Figure 4c).

### Effects of single vs. multiple species

To determine whether number of species included in the probiotic affected efficacy, single species probiotics were compared to multiple species probiotics. No significant difference between single and multiple species was observed (single species n = 51; RR = 0.73; 95% CI 0.68–0.79, multiple species n = 33; RR = 0.63; 95% CI 0.53–0.76) (Figure 4d).

### Publication Bias

**Figure 5:**
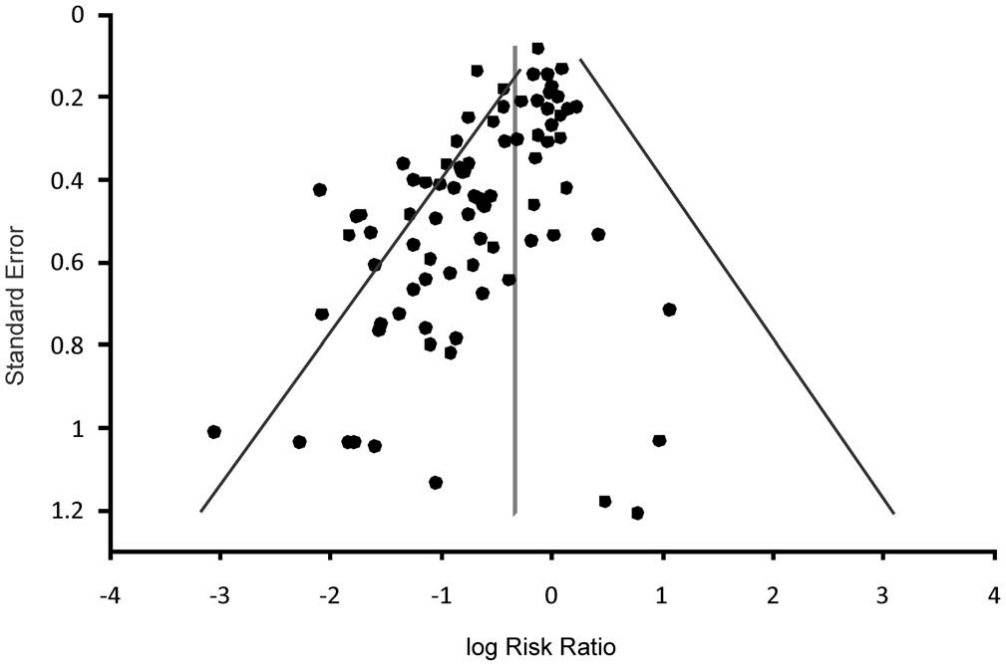
Funnel plot asymmetry used to determine publication bias. Log of the risk ratios were plotted against the standard error of the risk ratio of each study to identify asymmetry in the distribution of trials. Gaps in the funnel plot suggest potential publication bias. The synthesis estimate and the 0.01 limit are shown to distinguish asymmetry.

The funnel plot had an asymmetrical distribution (Figure 5). The Egger regression test (p>0.0001) and the Begg rank correlation test (p>0.0001) showed significant evidence of publication bias. However, using the fail-safe N method, we estimated that a total of 3,657 missing studies that would bring the p-value greater than alpha, were required to overturn the current results. The trim and fill method was used to correct for publication bias and yielded an overall effect size of 0.73 (95% CI 0.63–0.83), compared to the uncorrected overall effect size of 0.58 (95% CI 0.51–0.65).

## Discussion

Across all 11 probiotic species and the eight different gastrointestinal diseases we found a significant effect of probiotics on prevention and treatment of gastrointestinal disease with a RR = 0.58 (95% CI 0.51–0.65). Traveler's Diarrhea and Necrotizing Enterocolitis and the species *L. acidophilus*, *L. plantarum,* and *B. infantis* showed no efficacy. Previous meta-analyses that focused on efficacy of probiotics in the prevention or treatment of specific diseases have reported similar results. For example Johnston et al.. [13] reported a significant effect size (RR = 0.43 95% CI 0.25–0.75) for AAD disease, McFarland & Dublin [4] reported a significant effect size (RR = 0.78 95% CI 0.62–0.94) for IBS disease, and Elahi et al.. [18] reported a significant effect size (OR = 0.04 95% CI 0.01–0.14, p<0.0001) for Pouchitis.

Pouchitis (RR = 0.17 95% CI 0.10–0.30) had the greatest effect size of all the diseases analyzed and efficacy of probiotic treatment for Pouchitis was significantly different than TD, IBS, HPP, CDD, and AAD. Pouchitis occurs in 50% of patients with ulcerative colitis after undergoing ileal pouch anal anastomosis (IPAA) [46]. Pouchitis is caused by inflammation of the ileal pouch that is caused directly (toxins or invasions in the anal mucosa) or indirectly (changes in fatty acids and bile salts) [47]. A previous meta-analysis on the prevention of Pouchitis in patients that have undergone IPAA surgery showed that probiotics have a positive effect on the prevention of Pouchitis [18]. Recent evidence proposes that bacteria play a primary pathogenic role in causing inflammation in patients with Pouchitis [48]–[50]. Ruseler-van Embden [51] found that individuals with Pouchitis have fewer *Lactobacilli* and *Bifidobacterium.* Efficacy of probiotic treatment in Pouchitis was significantly higher than efficacy for TD, IBS, HPP, CDD, and AAD (Figure 3a). The high efficacy of probiotics we observed in the treatment of Pouchitis may be due to a number of factors related to trial design. For example, treatment of Pouchitis was limited to VSL #3 and LGG and the patients in Pouchitis trials were all adults.

ADD, ID, IBS, HPP, and CDD also had effect sizes that were significant. AAD is present when an individual has three or more abnormally loose bowel movements over a twenty-four hour period following antibiotic use [52]. HPP colonization is a common health problem, especially in developing countries [3], [53], that causes chronic low-level inflammation in the stomach lining and duodenum leading to the development of gastric and duodenal ulcers, as well as stomach cancer [54]. When treating HPP, patients are prescribed antibiotics which results in some individuals developing AAD. CDD, which is also associated with antibiotic use, occurs mostly in older adults, and usually only occurs in hospitalized patients [55]. Probiotics are thought to restore equilibrium in the gastrointestinal tract and protect against *C. difficile* colonization. AAD, HPP colonization, and CDD are associated with antibiotic treatment [3], [4]. Probiotics are thought to be a useful treatment in these diseases as they occur in part from alterations of the intestinal microflora [4]. ID is a type of acute diarrhea that impairs intestinal absorption of nutrients and can lead to malnutrition [2]. IBS leads to abdominal pain, bloating, diarrhoea, constipation, and flatulence due to motor and sensory dysfunction of the gastrointestinal tract [4].

Our observation of significant efficacy for ADD, ID, IBS, HPP, and CDD support other recent meta-analyses on specific GIT diseases. McFarland [1] showed that AAD is preventable by probiotics; McFarland & Dublin [4] demonstrated that probiotics have a significant effect on the improvement of IBS, and Tong et al.. [3] suggested that probiotics could be effective in increasing eradication rates of anti-*H. pylori* therapy. Although in the latter study Tong et al.. [3] showed that *H. pylori* eradication rates were 83.6% for patients with probiotics and 74.8% for patients without, and thus suggested that larger trials were needed to confirm a significant effect. Probiotics have also been shown to have significant efficacy for CDD [1]. Our result for ID represents the first meta-analysis of probiotic use in ID treatment as only single trials (e.g. [56]) have previously been conducted.

Two of the GIT diseases considered here, Traveller's diarrhea (TD) and Necrotizing Enterocolitis (NEC), showed no significant effect of probiotics. TD is a type of acute diarrhea that impairs intestinal absorption of nutrients and can lead to malnutrition [2]. Traveller's diarrhea is typically caused by an amoeba [57] and is treated with antibiotics that also lead to diarrhea. Our results support previous studies by Pozo-Olano et al.. [58] and Katelaris et al.. [59] who both found probiotics to have no effect in people suffering with traveller's diarrhea. In contrast, Hilton et al.. [27] showed that LGG can reduce the risk of developing diarrhea by 3.9% per day.

NEC was the only other gastrointestinal disease that did not show a significant effect for treatment with probiotics. NEC is a gastrointestinal disease that is a major issue in preterm (<28 weeks' gestation) neonates and involves infection and inflammation that causes destruction of the bowel or part of the bowel [17]. NEC only affects 1% to 5% of neonatal intensive care unit (NICU) admissions, but it is common worldwide and is the most serious disorder among hospitalized preterm infants. A possible explanation is that NEC occurs mostly in infants and infants do not have their immune system or their microbial communities fully established [60]. Our results, based on ten studies, differ from those of Deshpande et al.. [17] who showed that probiotics significantly reduce the risk of NEC (RR = 0.36 95% CI 0.20–0.65) in preterm neonates, however they suggested that probiotics needed to be assessed in larger trails in order to determine their short and long term effects in the treatment of NEC. Our meta-analysis improves on their meta-analysis by adding three studies.

We initially hypothesized that probiotic use might be more efficacious in some broad types of GI diseases than in others due to the mechanisms of action of the disease. Specifically, that there might be differences in efficacy related to diarrheal production versus inflammation or destruction of tissue, verses abdominal pain, flatulence and irregular bowel movements (IBS). We found no support for this hypothesis. AAD, CDD, ID, and TD are related to diarrhea and NEC, Pouch and HPP are related to inflammation/destruction of tissue. IBS is characterized by abdominal pain, increased flatulence and irregular bowel movements. None of these groups differed significantly in probiotic efficacy and all disease showed significant effects except for NEC and TD, which are related to inflammation and diarrhea respectively.

Previous studies have focused on the effect of one to two species of probiotics (e.g. [19], [61], [ and 62]) in the prevention of specific GI diseases. In this study, we analyzed efficacy across 11 probiotic species and their effects on GI diseases overall. Of the 11 probiotic species considered, VSL #3 (RR = 0.17 95% CI 0.09–0.33) and *C. butyricum* (RR = 0.18 95% CI 0.09–0.37) had the most significant effect sizes (Figure 3b). The high statistical efficacy for these species could be due to the small number of patients analyzed compared to the other species. For example, *C. butyricum* had 207 patients and VSL #3 had 116 patients which are small compared to LGG with 2782 patients. Higher efficacy for these species could also be related to their use in diseases that also showed high prevention/treatability with probiotics (e.g. AAD, HPP, Pouchitis), unlike species that are widely used across many different GI diseases, such as LGG, which is used in the prevention or treatment of TD, Pouchitis, CD, AAD, HPP, NEC, and IBS. LGG is used widely in clinical trials because of its beneficial effects on intestinal immunity [58]. Furthermore, LGG has shown to inhibit the growth of *Esherichia coli*, *Streptococci*, *C. difficile*, *Bacteriodes fragilis* and *Salmonella* by producing an antimicrobial substance [63]. *S. boulardii*, *E. faecum, B. lactis, LGG, L. casei,* and *L. acidophilis* combined with *B. infantis* also showed significant efficacy in the treatment and prevention of GI disease. Our results support recent findings by McFarland et al.. [64] who showed that *S. boulardii* prevented AAD and by Orrhage et al.. [65] who showed that the combination of *L. acidophilus* and *Bifidobacterium* reduced the faecal counts of clostridia in CDD.

The efficacy of probiotic treatment has been shown to be highly dependent on the genus, species, and even the strain of bacteria used [66]. For example, not all lactic acid bacteria have probiotic effects [67]. In the case of traveller's diarrhea, *acidophilus strain LB* have been found to be effective [68], whereas other strains of *Acidophilus* spp. have not [59]. Also, different probiotics may confer different degrees of benefit depending on the condition. For example, McFarland [1] found that 3 types of probiotics (*Saccharomyces boulardii, Lactobacillus rhamnosus GG* and probiotic mixtures) significantly reduced the development of AAD while, in the treatment of CDD only *Saccharomyces boulardii* was effective [1].

In our meta-analysis *L. acidophilis, L. plantarum*, and *B. infantis* did not have significant effect sizes, showing that they are not effective across all the GI diseases considered here. In this meta-analysis, all species of *L. acidophilus* were first analyzed together. This included strain LB, a common probiotic as well as unspecific strains. *L. acidophilus* (strain LB) a heat stabilized strain also known as LacteÂol Fort [68]. In some previous studies, LacteÂol Fort (*L. acidophilus LB*) has shown to be effective in the efficacy of acute diarrhea, reducing duration and severity [68], [69] and in IBS [28]. In the treatment of HPP, inactive *L. acidophilus* showed an in vitro inhibitory effect on the attachment of *H. pylori* to gastric epithelial cell lines [70]. In other studies *L. acidophilus* has not shown significant effects. For example, Katelaris et al.. [59] found no protection of TD with *L. acidophilus* and Witsell et al.. [71] found no effect of *L. acidophilus* on AAD. Our results suggest that when taken without other species, *L. acidophilus* is not significantly effective in preventing/treating GI disease (RR = 0.82 95% CI 0.47–1.43). This result may be due to analyzing the strains *L. acidophilus LB* and *L. acidophilus* together and strain dependency could have an effect on the efficacy of GI disease. When analyzed alone, *L. acidophilis* LB did show significant efficacy (RR = 0.40 95% CI 0.20–0.82) and *L. acidophilus* with no strain specified did not have a significant effect (RR = 1.17 95% CI 0.85–1.62). Future studies should compare and report effects of different strains of *L. acidophilus* on GI diseases. Sazawal et al.. [2] found that prevention did not vary significantly for the probiotic species *S. boulardii*, *LGG*, *L. acidophilus*, or *L. bulgaricus*. In our meta-analysis *L. plantarum and B. infantis* also showed no overall effect on GI disease. Similar negative results for *L. plantarum* have been previously shown in the treatment of IBS [37], [72], [73]. In contrast, *L. plantarum* has been shown to have efficacy in the prevention of CDD [74]. Additional studies across GI diseases need to be conducted to assess the specific diseases that respond to *L. plantarum*. We also found that *B. infantis* had no significant effect. There were very few trials available in the literature for this species (n = 3) [74] and additional studies should be done to test efficacy.

Probiotics may be given to patients as either single or multiple species. While some studies use one probiotic species e.g. *B. infantis* [75] others used multiple strains e.g. VSL #3 [26], [76], [77]. We found no significant difference between the efficacies of single or multiple species across all diseases (Figure 4d). Instead, as discussed above, the particular strain used is key to efficacy. Since most studies only included the species of probiotic (e.g. *L. acidophilus*) used, it is critical for future studies to include the exact probiotic strain.

Ontogenic changes in the composition of the gut microflora might also affect efficacy of probiotics [7], [78], [79], [80]. For example, in the colon of breast-fed infants prior to weaning, the fecal microbiota is dominated by *Bifidobacterium* spp., while in adults, *Bifidobacterium* spp. are only minor constituents [60]. Likewise, the colon of elderly individuals has lower proportions of *Veillonella* spp. and *Bifidobacteria* spp. relative to *Clostridia* spp., *Lactobacilli* spp., and *Enterobacteria* spp. [60]. Ontogenic differences such as these suggest that efficacy of probiotic-use and potentially overall outcome may differ based on age. A number of studies have shown that probiotic efficacy can differ in infants, children, and adults [81], [82], [83], [ and 84]. While the administration of probiotics to both infants and adults results in changes of the microflora present in the feces and the metabolic activity of the microflora [81], a number of studies have shown greater differences between adults and children in the composition of their fecal microflora communities than exist within a cohort [82]–[84], suggesting strong ontogenic differences.

Our results showed no difference in efficacy by age group with all age groups (infants, children, and adults) showing significant effect sizes with the use of probiotics for the prevention of GI disease (Figure 4a). Similar results have been reported by Tong et al.. [3] who showed that child and adult age group sub-analyses were both significant for HPP. Likewise, Sazawal et al.. [2] showed significant results for both children and adults for the prevention of acute diarrhea. A potential difference in the efficacy of probiotics based on patient age is an area where additional studies are needed. Very few trials have been conducted on infants (n = 9) or children (n = 14) relative to adults (n = 53). For example, Hoveyda et al.. [5] concluded that IBS was preventable for adults, but could not assess efficacy in children due to the lack of studies.

Another factor that has been previously considered in probiotic efficacy is dosage. Our results showed that three of the four dosage levels were significant in treating disease. Only the dose 1–9×10^11^, 10^12^ CFU/day, which was the largest treatment dose, did not show a significant effect size. However, this result was likely due to the smaller sample size (n = 7) relative to the sample sizes of the other doses (n = 20, 25, and 12), which contributed to a larger 95% CI. Whorwell et al.. [75] studied the probiotic *B. infantis* (strain 35624) at three different dosage strengths 10^6^, 10^8^, and 10^10^ and found 1×10^10^ CFU (for four weeks) was most effective. The dosages tested in the studies analyzed here all use dosages well above the minimum in commercial preparations, which typically contain more than 1 billion bacterial units [85]. Correct dosage for specific diseases has been an area of some debate. For example, Bezkorovainy [81] suggested that several billion organisms should be introduced into an organism as not all of the bacteria will reach target areas due to pH and salinity levels in the esophagus and stomach which can reduce colony size [60]. Our results suggest that dosage has relatively minor effects.

In the past, it has been suggested that the treatment length that patients received the probiotic could be a factor in the treatment or prevention of disease and longer studies should be implemented [4], [5]. To our knowledge, this is the only meta-analysis that has examined efficacy according to the length of treatment. Our results show no significant effect of treatment length on efficacy (Figure 4c). Taking probiotics for even a week is sufficient in preventing and treating GI disease.

Several limitations of this meta-analysis are important to consider in interpreting the results. An important limitation is heterogeneity in outcome assessment and study design, particularly for the overall analysis which includes different diseases, strains, dosages, age groups, treatment lengths, and outcomes. Although the studies were weighted by the number of patients, heterogeneity still exists; therefore a random effects model was performed. As in all meta-analyses, results need to be interpreted cautiously.

Another limitation is that publication bias was observed using the Begg and Egger method. Thus, a trim and fill method was used to correct for publication bias. A positive significant effect of probiotics was still observed. For future studies it would be helpful to perform fecal samples before and after treatment to distinguish the changes microbial communities, as well as specify adverse effects of the treatment and prevention. This data would help address the question of what changes probiotics are actually leading to in the microbial ecology of the GIT.

In conclusion, our meta-analysis containing 74 studies, 84 trials and 10,351 patients shows that in general, probiotics are beneficial in treatment and prevention of GI diseases. The only GI diseases where significant effect sizes were not observed were TD and NEC. This effect may be due to the low number of studies on these diseases, or in the TD case, the underlying mechanism of disease, which is often not bacterial. Of the 11 species or species mixtures only *L. acidophilus*, *L. plantarum* and *B. infantis* showed no efficacy, however, for *L. acidophilus*, it was found that the strain LB was highly effective. No differences in efficacy were observed for age group or length of treatment or for single vs. multiple species. The highest dosage considered (1–9×10^11^, 10^12^ CFU/day) did not show a significant effect size, however, due to the small sample size, this result may be spurious. When choosing probiotics, the type of disease (treated/prevented) and probiotic species (strain) used are the most important factors to take into consideration.

## Supporting Information

Materials S1 The supplementary material provided includes raw data on: ID (reference #), Author, Year, Event and group size (patients given probiotic), Event and group size (patients given control), patients disease, probiotic species, comparison of mutiple vs. single probiotics given, dose (1: 1–5×1010 cfu/day, 2: 1–9×109 cfu/day, 3: 1–5.5×106, 107, 108 cfu/day, 4: 1–9×1011, 1012 cfu/day), treatment lengths, age group, N (number of patients), rr (relative risk), 95% CI−, 95% CI+, z value, p value, weight % and quality score.
(XLS)

Appendix S1 Complete PRISMA search for Pubmed (1970 to 2011).
(DOCX)

Checklist S1 PRISMA Checklist.
(DOC)
