# Supplementary material for: A Meta-Analysis of Probiotic Efficacy for Gastrointestinal Diseases
Source: PLoS One. 2012 Apr 18;7(4):e34938. doi: 10.1371/journal.pone.0034938 (PMC3329544; doi:10.1371/journal.pone.0034938)
Supplement: Appendix S1 — Complete PRISMA search for Pubmed (1970 to 2011). (DOCX) [file pone.0034938.s002.docx]

**Appendix S1**

1. **Probiotics**
2. **Probiotic meta-analysis**
3. **Gastrointestinal Disease**
4. **Diarrhea**
5. **1 or 2 or 3 or 4**
6. **Helicobacter pylori**
7. **Pouchitis**
8. **Antibiotic Associated Diarrhea**
9. **Irritable Bowel Syndrome**
10. **Traveller’s Diarrhea**
11. **Clostridium difficile Disease**
12. **Necrotizing Enterocolitis**
13. **Infectious Diarrhea**
14. **6 or 7 or 8 or 9 or 10 or 11 or 12 or 13**
15. **Yogurt**
16. **5 or 14 or 5**
17. **5, 14 and 15**
18. ***Lactobacillus***
19. ***Bifidobacterium***
20. ***Saccharomyces***
21. ***Streptococcus***
22. ***Enterococcus***
23. **16 or 17 or 18 or 19 or 20**
24. **Randomized controlled trials**
25. **Controlled trials**
26. **Placebo**
27. **Control**
28. **5 and 24**
29. **5 and 25**
30. **5 and 26**
31. **5 and 27**
32. **5 or 23**
